# Procalcitonin to Predict Severity of Acute Cholangitis and Need for Urgent Biliary Decompression: Systematic Scoping Review

**DOI:** 10.3390/jcm11051155

**Published:** 2022-02-22

**Authors:** Krixie Silangcruz, Yoshito Nishimura, Torrey Czech, Nobuhiko Kimura, James Yess

**Affiliations:** 1 Department of Internal Medicine, University of Hawai’i, Honolulu, HI 96813, USA; kvs309@hawaii.edu (K.S.); tczech@hawaii.edu (T.C.); kimurano@hawaii.edu (N.K.); jyess@queens.org (J.Y.); 2 Department of General Medicine, Okayama University Graduate School of Medicine, Dentistry and Pharmaceutical Sciences, Okayama 7008558, Japan

**Keywords:** procalcitonin, acute cholangitis, scoping review, systematic review

## Abstract

Serum procalcitonin (PCT) has been reported as a potential biomarker to predict the severity of acute cholangitis (AC) or the need for urgent biliary decompression. This study aimed to identify and summarize the existing research about serum PCT and the severity of AC, and to find gaps towards which future studies can be targeted. Following the PRISMA extension for scoping reviews, MEDLINE, EMBASE, and Google Scholar were searched for all peer-reviewed articles with relevant keywords including “cholangitis” and “procalcitonin” from their inception to 13 July 2021. We identified six studies. All the studies employed a case-control design and aimed to evaluate the usefulness of serum PCT to predict the severity of AC with key identified outcomes. While the potential cut-off values of serum PCT for severe AC ranged from 1.8–3.1 ng/mL, studies used different severity criteria and the definition of urgent biliary decompression. No studies proposed cut-off PCT values for the need for urgent biliary decompression. This scoping review identified the current level of evidence regarding the usefulness of serum PCT in assessing the severity of AC. Further clinical research is warranted with a focus on standardized outcome measures employing prospective or experimental designs.

## 1. Introduction

Acute cholangitis (AC) is a medical emergency and systemic condition due to biliary infection and obstruction with an associated high mortality rate [1,2]. Before advancements in critical care and the decompression of the biliary duct system, the mortality of AC was reported to be over 50% [2]. Subsequent to 1980, the mortality rates of AC ranged from 10–30%, with multiorgan failure noted to be the cause of death [3]. The majority of cases of AC are due to biliary duct stones. The variety of additional etiologies underscores a wide variety of risk factors that influence mortality [3,4].

Procalcitonin (PCT), a 116 amino acid peptide precursor of calcitonin, was initially thought to help identify sepsis patients and was later associated with bacterial infection [5]. However, its role in disease severity prediction remains unclear [6], with a reported sensitivity and specificity of PCT to predict septic shock of any causes by 63% and 65%, respectively [7]. Thus, the use of PCT in the diagnosis of sepsis remains a topic of contentious debate [8].

Various studies have found PCT to be correlated with the disease severity of AC. The Tokyo Guidelines 2018 (TG18) for AC discussed the utility of PCT as a parameter for severity assessment, stating that there was level D (very low-quality) evidence, and it might be an area of future research [9]. So far, small-scale studies have been conducted to examine the usefulness of serum PCT values to predict the severity of AC or the need for urgent biliary decompression, using either Tokyo Guidelines 2007 (TG07) and Tokyo Guidelines 2013 (TG13) [10,11,12,13,14,15]. However, to date, no systematic reviews are available to analyze the evidence from studies across different settings and clinical outcomes to identify the trends of PCT in AC or limitations with current literature to generate recommendations in this area for future research.

The objective of this study is to scope the states of research to determine the relationship between serum PCT levels and AC disease severity, the trends correlating PCT levels to different hierarchical levels of intervention as well as the need for urgent biliary decompression to identify directions for future research in this area.

## 2. Materials and Methods

### 2.1. Study Design

This is a systematic scoping review conducted in accordance with the Preferred Reporting Items for Systematic Reviews and Meta-Analyses (PRISMA) extension for scoping reviews (PRISMA-ScR) [16,17]. See Supplementary File S1 for PRISMA-ScR Checklist of the present study.

### 2.2. Search Strategy

We searched MEDLINE, EMBASE, and Google Scholar for all peer-reviewed articles and conference abstracts from inception to 13 July 2021. No filters for study design and language were used. A manual screening for additional pertinent articles was done using the reference lists of all articles that met the eligibility criteria. The search strategy involved relevant keywords, including “cholangitis” and “procalcitonin.” The search was conducted by two authors (YN and NK) independently. See Supplementary File S2 for detailed search terms.

### 2.3. Eligibility Criteria

The criteria for the inclusion of articles are the following:

(1) Peer-reviewed articles evaluating the relationship between serum procalcitonin levels and severity of acute cholangitis or need for biliary decompression;
(2) Randomized controlled trials (RCTs), case-control studies, cohort studies (prospective or retrospective), cross-sectional studies, and case series in adult patients.

The exclusion criteria included the following:

(1) Qualitative studies, review articles, case reports, and commentaries;
(2) Conference abstracts;
(3) Studies involving pediatric or obstetric patients.

Articles selected for full-text assessment were assessed independently by YN and NK using EndNote 20 reference management software. Articles considered eligible were then evaluated in full length with the inclusion and exclusion criteria.

### 2.4. Data Extraction

A standardized data collection form that followed the PRISMA and Cochrane Collaboration guidelines for systematic reviews was used to obtain the following information from each study: title, name of authors, year of publication, country of origin, study characteristics, target outcome, aims, study and comparative groups, key findings, and limitations.

## 3. Results

### 3.1. Search Results and Study Selection

**Figure 1:**
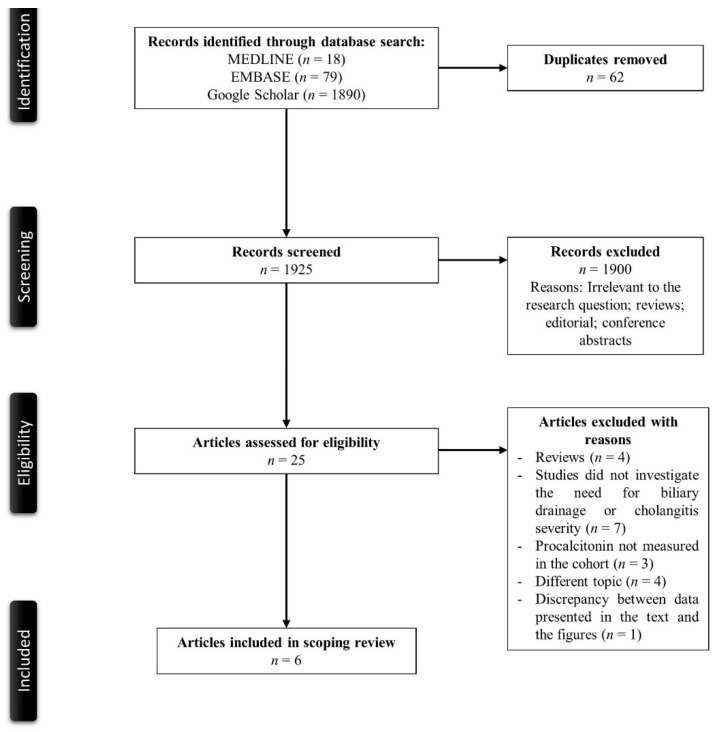
PRISMA (Preferred Reporting Items for Systematic Reviews and Meta-Analyses) flowchart of the search strategy.

Figure 1 shows a PRISMA flow diagram that depicts the process of identification, screening, eligibility, and inclusion or exclusion of the studies. The initial search of MEDLINE, EMBASE and Google Scholar yielded 1987 articles. The 62 duplicate studies were removed, followed by the elimination of 1900 articles that were either irrelevant to our study, review articles, editorial articles, or conference abstracts with title and abstract screening. Subsequently, 25 articles underwent a full-text review. Of these articles, 19 articles, because they were review articles, did not investigate the need for biliary drainage or cholangitis severity as an outcome, did not measure procalcitonin in the participants, or were not studies about procalcitonin and acute cholangitis. One study was also excluded because of the discrepancy between the data presented in the main text and the figures.

### 3.2. Description of the Included Studies

A total of six studies met our eligibility criteria for the scoping review [10,11,12,14,18,19]. The main characteristics of the included studies are described in Table 1. Five were retrospective case-control studies, while one study [12] used a prospective case-control design. All the studies were from Asia (Japan, *n* = 4; South Korea, *n* = 1; China. *n* = 1). Sample sizes varied from 58 to 213 participants. All studies aimed to evaluate the usefulness of PCT to predict the severity of AC and were based on data from a single center.

### 3.3. Outcomes

Four studies also assessed blood culture positivity among participants [11,12,14,19]. The number of patients who went through urgent biliary decompression was reported in three studies [12,14,19]. Of the three studies, the studies by Lee et al. and Umefune et al. reported timing of biliary decompression [12,14]. Four studies assessed positivity of blood culture in patients with AC [11,12,14,19].

### 3.4. Severity of Acute Cholangitis

Findings related to serum PCT levels and the severity of AC or other pertinent outcomes are summarized in Table 2. Except for one study [10], TG13 Guidelines were used as the severity criteria of AC. There was a significant variation in the proportion of patients with severe AC from 7/122 (5.7%) [19] to 25/110 (22.7%) [11]. All studies reported that serum PCT levels were significantly higher in those with severe AC than those with mild or moderate AC, although the median serum PCT level differed considerably between the included articles. Area under the receiver operator characteristic (AU-ROC) of serum PCT for severe AC varied from 0.75 (95% confidence interval [CI] 0.63–0.87) [11] from 0.90 (95%CI 0.85–0.96) [12]. Except for one study [19], the potential cut-off values of serum PCT for severe AC were proposed, which ranged from 1.76 ng/mL (sensitivity 84.6%, specificity 62.4%) from 3.1 ng/mL (sensitivity 80.8%, specificity 84.6%) [10] using the Youden Index method, with three studies that reported similar cut-off values (Umefune et al. 2.2 ng/mL, Shinya et al. 2.33 ng/mL, Lyu et al. 2.38 ng/mL).

### 3.5. Sensitivity Analysis

Although some studies included the term “biliary drainage” or “biliary decompression” [11,14], no studies proposed potential cut-off values for the need for urgent or emergent biliary decompression. The surrogate of the need for urgent or emergent biliary decompression varied considerably among the studies. Korekawa et al. [11] used thrombocytopenia as the surrogate and suggested that the cut-off serum PCT to predict lower platelet counts (1.3 ng/mL) might also be helpful to determine those who may need urgent biliary decompression. Shinya et al. [11] proposed a cut-off PCT for the presence of purulent bile juice on biliary decompression (3.2 ng/mL) as the surrogate. Other studies used the severity of AC as the surrogate for the need for urgent biliary decompression. Of note, the definitions of urgent or emergent decompression were different among the articles. Korekawa et al. defined “emergent endoscopic retrograde cholangiopancreatography (ERCP)” as “the procedures done within 24 h after admission”, while Umefune et al. defined urgent and early biliary drainage as “biliary drainage six hours and 12 h after admission”, respectively. Other studies did not specify the term of early, urgent, or emergent biliary decompression.

## 4. Discussion

In this scoping review, we identified six primary studies related to serum PCT levels and the severity of AC or other pertinent outcomes such as blood culture positivity, progression to septic shock, need, and timing of biliary decompression, or biliary fluid character on ERCP. All studies reported that serum PCT levels were significantly higher in those with severe AC than those with mild or moderate diseases, although the median serum PCT levels were considerably different among the included articles.

AC requires appropriate treatment in the early phase because severe AC may result in death if no early appropriate medical care is provided. Historically, TG13 proposed management bundles of AC, and TG18 re-defined the management bundles and mentioned the potential utility of serum PCT in predicting the severity of AC [15,20]. These guidelines classified the severity of AC into three grades; mild (grade I), moderate (grade II), and severe (grade III) [21]. Supplementary File S3 summarizes the TG18/TG13 severity grading of AC. Except for the redefinition of the management bundles, no changes were made in the severity grading in TG18 compared to TG13. In particular, the TG18 severity grading criteria for AC are essential for predicting prognosis and determining a treatment strategy by identifying patients requiring early biliary drainage, although its ability to identify those who need the procedure is limited [22]. As noted above, there was very low-quality evidence suggesting the utility of PCT as a parameter for the severity assessment of AC when TG18 was proposed.

The present study results showed that previous literature had a certain degree of consistency regarding a potential serum PCT cut-off value for severe AC (ranging from approximately 1.8 ng/mL to 3.1 ng/mL, except for the one by Lee et al., likely because they included considerably more patients with moderate AC) with satisfactory AU-ROC. However, all the studies had different limitations, as described in Table 1. While the proposed serum PCT cut-off values for severe AC were similar, median PCT values of either moderate or severe AC differed considerably between the included studies. Of note, no studies proposed cut-off values to proceed with urgent biliary decompression, and the severity of AC was used as a surrogate of the need for the procedure. As far as the timing of PCT measurement was concerned, four of the six studies, Hamano, et al., Umefune, et al., Lee et al., and Lyu, et al. measured PCT level at the time of admission. Shinya, et al. performed measurement of PCT at the time of diagnosis of AC. Korekawa et al. did not mention the time point when they checked PCT.

In addition, there were inconsistencies in the severity criteria used (Hamano et al. used TG07 and the others employed TG13) and the definition of either “urgent” or “emergent” decompression. For example, Umefune et al. defined urgent biliary drainage as “within 6 h from admission”, while Lee et al. classified patients according to the timing of biliary drainage; within 24 h, from 24 to 48 h, or after 48 h from a hospital visit. The differences in the guidelines used and the outcome measurements negatively affect the level of evidence.

Despite the downsides discussed above, elevated serum PCT still may be helpful to triage patients with AC from different etiology, such as malignancy. In particular, those with malignancy often have leukocytosis or serum inflammatory markers at their baseline, making it difficult to discriminate whether they suffer from acute infections or not. Most of the patient representatives in the studies had AC due to choledocholithiasis. Some other etiologies, but with mostly small percentages, include benign stricture from chronic pancreatitis, malignant stricture from cholangiocarcinoma, pancreatic head cancer, and gall bladder cancer with bile duct invasion. None of the six studies performed a subanalysis on the etiologies of AC and PCT values. Thus, a focus of future studies may need to be on the relationship between serum PCT and severity of AC in different etiologies.

It is important to note that the limitations of the studies included were that all of the currently available research were case-control studies (five retrospective and one prospective) with small sample sizes. In addition, the considerable heterogeneity of the basic demographics among the studies needs to be noted. All the studies were conducted in Asian countries, including Japan, South Korea, and China. Further, the etiology of AC may be different; for instance, 9.8% of patients in the study by Lee et al. had malignant biliary strictures, while Shinya et al. included only patients with choledocholithiasis. All the factors limit the generalizability of the results. Furthermore, due to the urgent need for evidence on this topic and limited time, we did not contact authors to clarify the details of the data described in the literature. Next, we only included peer-reviewed articles in this scoping review. Thus, non-peer-reviewed articles or conference abstracts, which might have been useful, were excluded from the study.

Given the limitations, future studies may need adequate power with a prospective design and standardized outcome measurements, using the same severity criteria and timing of biliary decompression, including patients with diverse etiologies such as malignancy. To the best of our knowledge, however, there has been no scoping review to investigate the utility of serum PCT to predict the severity of AC or the need for biliary decompression.

## 5. Conclusions

In conclusion, this scoping review identified that the current body of evidence regarding the usefulness of serum PCT in assessing the severity of AC, and potential cut-off values for the need for urgent biliary decompression, remains scarce. However, future studies are warranted to see whether elevated PCT may help triage those who benefit from emergent interventions among patients with AC due to biliary obstructions from different etiologies.

## Figures and Tables

**Table 1:** Main characteristics of the included studies in the scoping reviews.

Author, Year, Country	Study Type	Aim	Outcome	Population	Comparative Groups	Key Findings	Limitations
Hamano et al., 2013 Japan [10]	CC	To evaluate the usefulness of PCT for AC severity assessment	Serum PCT value	Severe AC (*n* = 26)	Mild (*n* = 39) and moderate (*n* = 94) AC	PCT was significantly higher in patients with severe AC than those with mild or moderate (*p* < 0.0001)	No cut-off for early/urgent biliary drainage. Other infections not excluded at the baseline
Shinya et al., 2014 Japan [11]	CC	To evaluate the correlation of AC severity and serum PCT	Serum PCT value Blood culture positivity	Severe AC (*n* = 25)	Mild (*n* = 22) and moderate (*n* = 63) AC	PCT was significantly higher in patients with severe AC than those with mild AC (*p* < 0.001) Those with positive blood culture (*n* = 13) had significantly higher PCT than those with negative blood culture (*n* = 59; *p* = 0.001) In patients with mild or moderate AC, those with purulent bile juice on ERCP (*n* = 24) had significantly higher PCT than those with normal bile juice from the duodenal papilla (*n* = 48; *p* < 0.001)	All included patients had AC due to choledocholithiasis Blood culture was obtained only in 72 patients
Umefune et al., 2017, Japan [12]	CC (prospective)	To evaluate the correlation between serum PCT on admission and severity of AC	AC severity (TG13) The ability of PCT to predict severe AC due to choledocholithiasis/stent occlusion/positive blood culture Timing of biliary decompression	Severe AC (*n* = 29)	Mild (*n* = 108) and moderate (*n* = 76) AC	Patients with multiple causes of AC included (*n* = 107 with stent occlusion, *n* = 82 with choledocholithiasis, *n* = 24 with other) PCT was significantly higher in patients with severe AC than those with mild AC (*p* < 0.0001) Those with positive blood culture (*n* = 58) had significantly higher PCT than those with negative blood culture (*n* = 112; *p* < 0.0001) Those with severe AC had urgent biliary drainage (within 6 h from admission) more frequently than those with mild or moderate AC (*p* = 0.0096)	Small sample size to limit the robustness of secondary analyses Data from a single tertiary care center causing a bias in the characteristics of the enrolled patients
Lee et al., 2018, South Korea [14]	CC	To determine the association of serum PCT with AC severity and clinical deterioration	Positive blood culture Method of biliary decompression Timing of biliary decompression	Severe AC (*n* = 26)	Mild (*n* = 39) and moderate (*n* = 139) AC	PCT was significantly higher in patients with severe AC than those with mild or moderate (*p* = 0.001) Patients who progressed to septic shock had significantly higher PCT than others (*p* = 0.040) No significant difference in timing of biliary decompression between groups (*p* = 0.341) Patients with positive blood culture (*n* = 65) had significantly higher PCT than those with negative blood culture (*p* = 0.001)	Considerable portion of patients with malignant stricture (*n* = 20, 9.8%) 95%CI for IQR not listed Only 6 (2.9%) patients had septic shock
Lyu et al., 2014, China [18]	CC	To evaluate the role of PCT to assess the severity of AC	Serum PCT value	Severe AC (*n* = 28)	Mild (*n* = 70) and moderate (*n* = 49) AC	PCT was significantly higher in patients with severe AC than those with mild or moderate (*p* < 0.05)	Retrospective data from a single center
Korekawa et al., 2020, Japan [19]	CC	To evaluate the usefulness of PCT to diagnose AC, and to determine a management plan	Serum PCT value Urgent biliary decompression Blood culture positivity	Severe AC (*n* = 7)	Mild (*n* = 57) and moderate (*n* = 58) AC	PCT was significantly higher in patients with severe AC than those with mild or moderate (*p* < 0.0001) Platelets were significantly lower in those with PCT of more than 1.3 ng/mL	Only seven severe AC patients were included

Abbreviations: AC, acute cholangitis; CC, case control; ERCP, endoscopic retrograde cholangiopancreatography; IQR, interquartile range; PCT, procalcitonin; TG, Tokyo Guidelines.

**Table 2:** Key findings related to serum procalcitonin levels in the included studies.

Author, Year, Country	Severity Criteria Used	PCT–Median (ng/mL)			Cut-Off PCT for Urgent Biliary Decompression (ng/mL)	Cut-Off PCT for Severe AC (ng/mL)	AU-ROC of PCT	Comments
		Mild AC	Moderate AC	Severe AC				
Hamano et al., 2013 Japan [10]	TG07	0.08 (IQR; 0.04–0.18)	0.37 (IQR; 0.15–1.85)	5.56 (IQR; 3.59–25.89)	N/A	3.1; sensitivity 80.8%, specificity 84.6%, OR 23.1 [95%CI 8.0–70.2]	Severe vs. mild to moderate 0.86 (95%CI 0.78–0.92)	N/A
Shinya et al., 2014 Japan [11]	TG13	0.2 (IQR; 0.1–0.7)	0.7 (IQR; 0.2–2.7)	6.8 (IQR; 0.5–48.3)	N/A (cut-off PCT to predict those with purulent bile juice from the duodenal papilla was noted)	2.33; sensitivity 64.0%, specificity 78.0%	Severe AC 0.75 (95%CI 0.63–0.87) For purulent bile juice 0.77 (95%CI 0.64–0.89)	Median PCT of those with purulent bile juice: 7.9 ng/mL (IQR; 1.2–37.7) vs. 0.6 (IQR; 0.2–2.9) in those with normal bile juice Cut-off PCT for purulent bile juice: 3.2 ng/mL (sensitivity 67.0%, specificity 79.0%)
Umefune et al., 2017 Japan [12]	TG13	0.45 (IQR; 0.22–1.69)	1.25 (IQR; 0.41–4.18)	19.51 (IQR; 4.41–53.36)	N/A	2.2; sensitivity 97.0%, specificity 73.0%	Severe AC 0.90 (95%CI 0.85–0.96); better than WBC or CRP	Median PCT of those with positive blood culture: 4.71 ng/mL (IQR, 0.87–16.96) vs. 0.65 ng/mL (IQR, 0.25–2.26) in those with negative blood culture
Lee et al., 2018, South Korea [14]	TG13	0.22 (IQR; 0.52)	1.35 (IQR; 4.67)	9.41 (IQR, 53.69)	N/A	1.76 (for severe AC or septic shock); sensitivity 84.6%, specificity 62.4% 3.77; sensitivity 80.0%, specificity 74.0%	Severe vs. mild to moderate 0.778 (95%CI 0.680–0.876)	Median PCT of those with positive blood culture: 3.25 ng/mL (IQR, 8.86) vs. 0.62 ng/mL (IQR, 3.78) in those with negative blood culture Median PCT of those progressed to septic shock: 9.11 ng/mL (IQR, 18.52) vs. 0.89 ng/mL (IQR, 4.34) in others
Lyu et al., 2014, China [18]	TG13	0.166 (mean; SD ± 0.033)	0.349 (mean; SD ± 0.046)	0.759 (mean; SD ± 0.029)	N/A	2.38 (for severe vs. moderate AC); sensitivity 78.9%, specificity 73.7%	N/A	N/A
Korekawa et al., 2020, Japan [19]	TG13	0.9 (IQR; 0.1–1.1)	9.9 (IQR; 1.2–15.9)	37.8 (IQR; 20.3–54.9)	N/A (cut-off PCT to predict those with lower platelets, who might be complicated with DIC and potential candidates for urgent biliary decompression)	N/A	Severe and moderate vs. mild 0.89 (95%CI 0.84–0.95)	Median PCT of those with positive blood culture: 12.7 ng/mL (IQR, 0.45–19.8) vs. 4.6 ng/mL (IQR, 0.2–4.9) in those with negative blood culture Cut-off PCT for positive blood culture: 1.3 ng/mL (sensitivity 67.0%, specificity 55.0%) Those with PCT > 1.3 ng/mL had significantly lower platelets than others (*p* < 0.0001)

Abbreviations: AC, acute cholangitis; AU-ROC, area under the receiver operator characteristic; CI, confidence interval; CRP, C-reactive protein; DIC, disseminated intravascular coagulopathy; IQR, interquartile range; OR, odds ratio; PCT, procalcitonin; SD, standard deviation; TG, Tokyo Guidelines; WBC, white blood cell.

## Data Availability

The datasets generated and analyzed during the current study are available from the corresponding author on reasonable request.

## Supplementary Materials

The following supporting information can be downloaded at: https://www.mdpi.com/article/10.3390/jcm11051155/s1.
