# Supplementary material for: Procalcitonin to Predict Severity of Acute Cholangitis and Need for Urgent Biliary Decompression: Systematic Scoping Review
Source: J Clin Med. 2022 Feb 22;11(5):1155. doi: 10.3390/jcm11051155 (PMC8910914; doi:10.3390/jcm11051155)
Supplement: Supplementary file 1 [file jcm-11-01155-s001.zip › jcm-1514695-supplementary/jcm-1514694-Supplementary File S2.pdf]

Medline

(“cholangitis”[MeSH Terms] OR “Cholangitis”[All Fields]) AND (“procalcitonin”[MeSH Terms] OR “Procalcitonin”[All Fields])

EMBASE

(‘cholangitis’/exp OR cholangitis) AND (‘procalcitonin’/exp OR procalcitonin)

Google Scholar

Cholangitis AND Procalcitonin
