# Supplementary material for: Procalcitonin to Predict Severity of Acute Cholangitis and Need for Urgent Biliary Decompression: Systematic Scoping Review
Source: J Clin Med. 2022 Feb 22;11(5):1155. doi: 10.3390/jcm11051155 (PMC8910914; doi:10.3390/jcm11051155)
Supplement: Supplementary file 1 [file jcm-11-01155-s001.zip › jcm-1514695-supplementary/jcm-1514694-Supplementary File S3.pdf]

TG18/TG13 Severity Grading of Acute Cholangitis

**Grading**		
(A) Cardiovascular dysfunction<br>Hypotension requiring dopamine (≥5 μg/kg per min or any dose of norepinephrine)	Yes	No
(A) Neurological dysfunction: Disturbance of consciousness	Yes	No
(A) Respiratory dysfunction (PaO_2/FiO_2 ratio <300)	Yes	No
(A) Renal dysfunction: Oliguria or creatinine >2.0 mg/dL	Yes	No
(A) Hepatic dysfunction (INR >1.5)	Yes	No
(A) Hematological dysfunction (Platelet count <100,000/mm^3)	Yes	No
(B) Abnormal WBC count (>12,000/mm^3 or <4,000/mm^3)	Yes	No
(B) High fever (≥39°C/102.2°F)	Yes	No
(B) Age ≥75 years	Yes	No
(B) Hyperbilirubinemia (Total bilirubin ≥5 mg/dL)	Yes	No
(B) Hypoalbuminemia (<0.7 x upper limit of normal)	Yes	No
**Grade I Mild acute cholangitis: Not meeting the criteria for "severe" or "moderate"**<br>Recommendation: antibiotics and general supportive care; consider biliary drainage if no response to initial treatment		
**Grade II Moderate acute cholangitis: Any two of the "B" criteria**<br>Recommendation: antibiotics and general supportive care; early endoscopic or percutaneous transhepatic biliary drainage is indicated		
**Grade III Severe acute cholangitis: At least one of the "A" criteria**<br>Recommendation: initial treatment with antibiotics, urgent biliary drainage, appropriate respiratory/circulatory management		
